# On the Durability Performance of Two Adhesives to Be Used in Bonded Secondary Structures for Offshore Wind Installations

**DOI:** 10.3390/ma17102392

**Published:** 2024-05-16

**Authors:** Khaoula Idrissa, Aurélien Maurel-Pantel, Frédéric Lebon, Noamen Guermazi

**Affiliations:** 1 Aix-Marseille Université, CNRS, Centrale Marseille, LMA, CEDEX 13, 13453 Marseille, France; khawla.drissa@gmail.com (K.I.); maurel@lma.cnrs-mrs.fr (A.M.-P.); 2 LGME Laboratory, National Engineering School of Sfax, University of Sfax, Sfax B.P. 1173-3038, Tunisia; noamen.guermazi@enis.tn

**Keywords:** adhesives, thermal aging, hygrothermal aging, durability, mechanical properties

## Abstract

The development of offshore wind farms requires robust bonding solutions that can withstand harsh marine conditions for the easy integration of secondary structures. This paper investigates the durability performance of two adhesives: Sikadur 30 epoxy resin and Loctite UK 1351 B25 urethane-based adhesive for use in offshore wind environments. Tensile tests on adhesive samples and accelerated aging tests were carried out under a variety of temperatures and environmental conditions, including both dry and wet conditions. The long-term effects of aging on adhesive integrity are investigated by simulating the operational life of offshore installations. The evolution of mechanical properties, studied under accelerated aging conditions, provides an important indication of the longevity of structures under normal conditions. The results show significant differences in performance between the two adhesives, highlighting their suitability for specific operating parameters. It should also be noted that for both adhesives, their exposure to different environments (seawater, distilled water, humid climate) over a prolonged period showed that (i) Loctite adhesive has a slightly faster initial uptake than Sikadur adhesive, but the latter reaches an asymptotic plateau with a lower maximum absorption rate than Loctite adhesive; and (ii) a progressive deterioration in the tensile properties occurred following an exponential function. Therefore, aging behavior results showed a clear correlation with the Arrhenius law, providing a predictive tool for the aging process and the aging process of the two adhesives followed Arrhenius kinetics. Ultimately, the knowledge gained from this study is intended to inform best practice in the use of adhesives, thereby improving the reliability and sustainability of the offshore renewable energy infrastructure.

## 1. Introduction

The growing global demand for sustainable energy solutions has led to the rapid expansion of offshore wind farms, which have become key elements in the renewable energy landscape [1]. These installations, characterized by their massive scale and exposure to harsh marine environments, require technologies to ensure their longevity and structural integrity. At the heart of the wind turbine structure is the critical role played by bonded secondary structures in withstanding the harsh conditions of salt water, fluctuating temperatures, and long service life [2,3]. As offshore installations expand into deeper waters and more challenging environments, the importance of reliable bonding technology for secondary structures becomes increasingly important [4,5].

For these secondary structures, the choice of adhesive appears to be a critical issue given the wide range of conditions to which this material is subjected, including exposure to seawater, temperature fluctuations, and extended duty cycles [6,7]. This study will evaluate the performance of two adhesives specifically selected for their suitability in civil engineering and marine environments. The distinct performance differences observed between the two adhesives underscore their applicability within specific operational contexts, delineating their individual strengths and weaknesses under different conditions. For example, one adhesive’s superior initial take-up or resistance to certain environmental stressors may make it more suitable for applications where these factors predominate. Conversely, the increased long-term stability or durability demonstrated by another adhesive may make it preferable for installations where extended service life is a priority. By recognizing these differences, engineers and designers can make informed decisions on adhesive selection tailored to the precise requirements of their projects, thereby optimizing performance and ensuring structural longevity in offshore wind environments. The expected results will provide concrete information for the integration of bonded assemblies while ensuring the structural integrity of offshore wind farms [8,9]. Recent works have focused on the durability of adhesive bonds in marine composite structures, providing a nuanced overview of the challenges and advances in this evolving field [10,11,12].

Other studies have investigated the aging behavior of epoxy-based adhesives in marine environments, contributing to a better understanding of the long-term performance of adhesives subjected to these harsh conditions [3,12]. The effect of environmental influences on bonding in marine structures has been a major focus, providing crucial information on the longevity of adhesive joints subjected to the relentless forces of the marine environment [13].

Key references such as Andersen et al. [1], Brown et al. [14], and Gao et al. [9] provide valuable insights into the challenges and advances in adhesive technologies for offshore wind applications. Standards such as ASTM D638-14 [2,3] and ISO 3167:2013 [15] are highlights of the standardized test methods used in the field. Chen et al. [5] and Li et al. [16] investigated the effects of moisture absorption and the aging behavior of epoxy adhesives in marine environments. Kuang et al. [17] provided an overview of structural adhesives in offshore wind energy. In the studies of O’Connor et al. [18], Williams et al. [19], and Xu et al. [20], the focus was on the effects of the environment on bonding and assessing the durability of marine structures. The selection of these references is intended to provide a sound basis for this study, covering various aspects of bonding technology in the context of offshore wind turbines.

This study offers new insights into the behavior of Sikadur and Loctite adhesives under rigorous testing in different environmental conditions. In particular, the work of Pouyan Gand Noel M. H. [21] deals with the multi-scale modelling and life prediction of aged composites immersed in salt water. Their research provided a comprehensive understanding of how different composite materials age in such environments and valuable predictive insights into material degradation over time. In contrast, the study of Zike W and colleagues [22] investigated the long-term durability of BFRP/GFRP rods specifically in seawater and sea-sand concrete environments. This research provided insights into the specific performance of these reinforcing materials in marine environments, highlighting their durability and stability over long periods of time. In contrast, this study focuses on evaluating the performance of Sikadur and Loctite adhesives under various environmental conditions relevant to offshore wind environments. Through mechanical testing and accelerated aging simulations, including dry and wet conditions, the aim was to fully understand how these adhesives perform in real-world scenarios. This research contributes to the selection of materials for offshore wind turbines and advances the understanding of the role of adhesive technology in improving stability and durability in such environments.

## 2. Materials and Methods

The effectiveness of adhesive joints in offshore wind turbines is highly dependent on the choice of materials and the rigor of the testing methods. The materials studied are presented in detail. In addition, all the tests and methods used to characterize these adhesives are described.

### 2.1. Adhesive Materials

The adhesives studied in this work were Sikadur 30, manufactured by Sika Corporation, Bourget, France, and Loctite UK 1351 B25, manufactured by Henkel Adhesive Technologies, Düsseldorf, Germany. Both adhesives are intended to be used in bonded secondary structures for offshore wind turbines (balustrades, stairs, etc.).

#### 2.1.1. Sikadur Adhesive

Sikadur 30 is a two-component, thixotropic structural adhesive, based on epoxy resin and special fillers, designed for use in a temperature range from +8 °C to +35 °C. It has a glass transition temperature (Tg) of +52 °C (Sika France SAS, Paris, France).

The resin contains epoxy groups, reactive compounds essential for forming strong chemical bonds during curing. The hardener contains amines that react with the epoxy groups, initiating polymerization and creating a solid matrix with a reputation for durability.

According to the manufacturer, in terms of mechanical properties (Table 1), this adhesive has notable characteristics in several key areas.

Sikadur adhesive has excellent tensile strength, enabling it to withstand high loads. It also has good compressive strength, making it suitable for applications with concentrated loads. Its proven adhesion to a wide range of substrates ensures strong, durable bonds. Finally, its chemical resistance and thermal stability allow it to be used in a variety of environments. The absence of volatile solvents reinforces its environmental credentials.

All of these properties make Sikadur adhesive a good choice for structural applications that require reliable, long-lasting performance.

#### 2.1.2. Loctite Adhesive

The Loctite adhesive, UK 1351 B25, is a two-component, urethane-based adhesive which the manufacturer claims has excellent properties (Table 2).

Germanischer Lloyd (GL^®^)-approved Loctite adhesive is specially formulated for bonding epoxy-based composites.

According to the manufacturer, it is characterized by its fatigue resistance and its ability to maintain a reliable bond, even under difficult conditions. In addition, the Loctite adhesive offers resistance to crack propagation, making it a good choice for applications where durability and structural integrity are paramount. Its adaptability to a wide range of substrates makes it a versatile adhesive, while its ability to withstand harsh environments seems to reinforce its reputation as a reliable option in a variety of applications.

### 2.2. Sample Preparation Method

The adhesive sample preparation process is an important step in the evaluation of adhesive performance. This section describes the methodology used to prepare adhesive samples to be tested under various conditions.

#### 2.2.1. Mixing Preparation

In the case of Sikadur adhesive, the two components, epoxy resin and hardener, are accurately measured and then precisely mixed in the specified ratio. The resulting mixture is then applied evenly to the prepared surfaces of the mold (presented in the next section), taking care to maintain a constant thickness. Any air or vacuum trapped during application is carefully removed. The samples are then left in place for the specified polymerization time (7 days), to allow the adhesive to reach its maximum strength.

Similarly, with Loctite adhesive, the two components of the urethane-based adhesive are precisely proportioned and mixed. This mixture is applied to the designated areas of the mold, taking care to maintain an even thickness. Special attention is paid to the intimate contact between the adhesive and the mold through a controlled process. The adhesive is precisely placed in the mold and a heavy load is applied to exert considerable pressure. The samples are then cured under controlled conditions at room temperature to ensure optimum development of the adhesive’s properties. For this adhesive, the polymerization time was set at 24 h.

Both adhesives undergo the same rigorous process to produce standardized test specimens, ensuring accurate and reliable results in subsequent mechanical testing. This meticulous approach to specimen preparation is essential for ensuring the accurate and comparative evaluation of the performance characteristics of Sikadur and Loctite adhesives.

#### 2.2.2. Mold Fabrication

**Figure 1:**
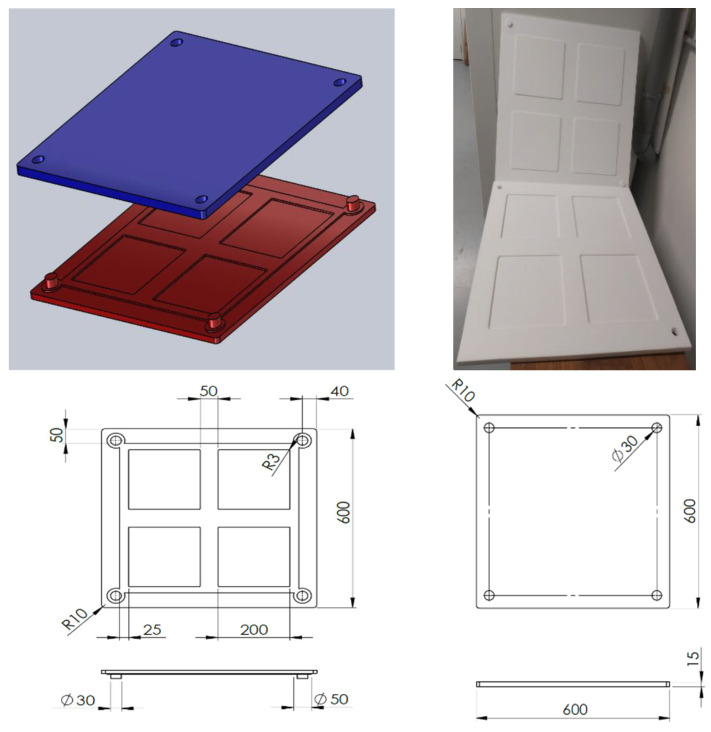
Geometry and dimensions of the Teflon mold.

Ensuring accurate and reliable test results requires a precise and controlled environment for adhesive application. The manufacture of the mold is therefore of great importance in the preparation of adhesive samples for experimental testing. The mold was produced using a precision machining process. First, the design specifications were transferred to the machining software, which then guided the cutting tools to form the molds from blocks of Teflon material. This process ensured that the dimensions and features of the mold were accurately reproduced in accordance with the design requirements. The Teflon mold (Figure 1), measuring 600 × 600 mm^2^ and 30 mm thick, was designed to produce uniform, well-defined adhesive plates. It consisted of two distinct parts: a lower plate and an upper plate, each 15 mm thick. Inside these plates were four precisely designed square cavities, measuring 200 × 200 mm^2^ and 3 mm thick (Figure 1).

**Figure 2:**
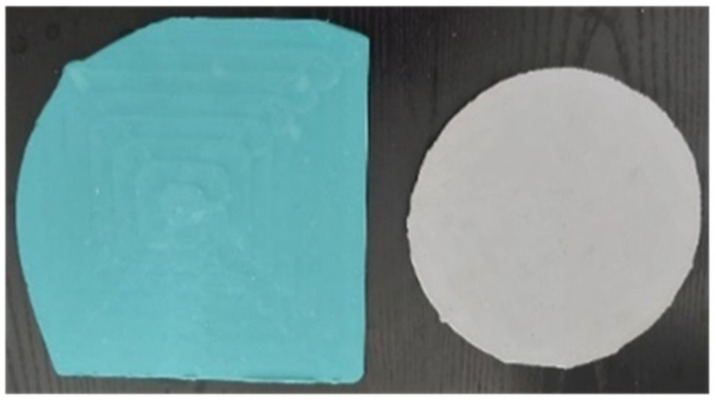
Examples of adhesive plates obtained through the molding process.

These cavities aid in the distribution of the adhesive, allowing the adhesive material to be evenly distributed. Once the cavities have been filled, the two mold plates were carefully closed to confine the adhesive to the specified areas. This process ensured the production of well-formed, uniform adhesive sheets (Figure 2), which were obtained by the molding process. These plates were then cut to obtain the adhesive samples for experimental testing.

Finally, the utilization of this Teflon mold guarantees the consistent production of test specimens, facilitating reliable and reproducible results across all tests. This allows reliable and reproducible results to be obtained in all tests. The careful manufacture of this mold is a key element in the preparation of adhesive samples in the form of plates for testing, as it ensures an accurate and reliable experimental results.

#### 2.2.3. Specimen Preparation

In this study, it is important to clarify that we only molded the adhesives for our experimental tests. The adhesive materials were accurately measured, mixed, and then applied to the designated areas of the mold. After curing, the resulting adhesive sheets were carefully cut to provide standardized test specimens. This method ensured consistent specimen preparation for subsequent mechanical testing.

Two different methods were used to cut the adhesive plates and produce the dumbbell specimens: milling and water jet cutting. Initially, the milling technique was used, using a specialized milling tool to achieve the precise cutting of the adhesive plates and produce the dumbbell specimens. This method was chosen because of its ability to accurately create intricate contours. However, to speed up the process, a transition was made to waterjet cutting. Waterjet cutting was chosen for its ability to produce clean and accurate cuts without generating excessive heat or material deformation.

**Figure 3:**
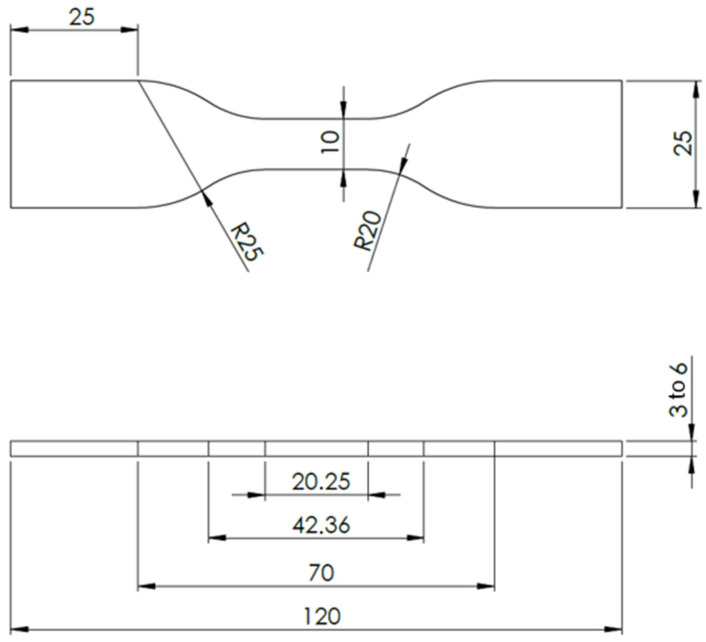
Geometry of the adhesive specimens used in the tensile tests (all units in mm).

The use of these two complementary methods made it possible to produce adhesive samples to the required specifications. The adhesive samples obtained and subjected to tests have a specific geometry, shown in Figure 3.

### 2.3. Experimental Protocol of Aging

**Figure 4:**
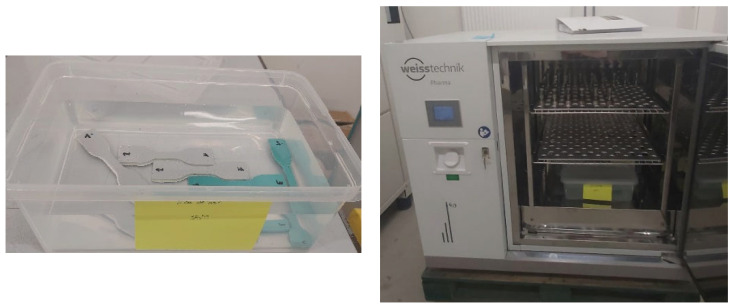
Testing container (on the (**left**)) and climate chamber/oven (on the (**right**)).

To investigate the durability performance of the two adhesives under different environmental conditions, accelerated aging tests were carried out by exposing the adhesive samples to different environments and temperatures. Special containers with covers were designed and used to hold the test specimens, providing a controlled test environment (Figure 4, left).

In addition, the adhesive samples were exposed to three different environments: a humid environment, distilled water, and seawater. Each of these environments simulated the realistic environmental conditions that adhesives may encounter in offshore wind turbines in a marine environment. The tests were therefore carried out at three different temperatures: ambient (22 °C), 35 °C, and 42 °C.

The adhesive samples were then placed in special ovens (Figure 4, right). This allowed the precise control of the environmental and thermal conditions throughout the aging test. This approach was important in accelerating the aging process and assessing the resistance of the adhesive materials under extreme conditions. This tool can also be effective in better understanding the long-term resistance of adhesive materials in marine environments.

The samples were aged for different lengths of time depending on the type of adhesive: the Sikadur samples were aged for up to 163 days, while the Loctite samples were aged for up to 231 days. These extended aging periods were chosen to thoroughly evaluate the long-term behavior and durability of the adhesives under different environmental conditions, providing valuable insight into their performance over time. In addition, by subjecting the samples to accelerated aging, we were able to speed up the prediction of their behavior over longer periods, making it easier to assess their durability and stability in marine environments.

#### 2.3.1. Thermal Aging

As mentioned above, thermal aging was carried out at three temperatures, such as room temperature (22 °C), 35 °C, and 42 °C. The selection of accelerated aging temperatures was based on several considerations. Firstly, the aim was to ensure that the selected temperatures were within a range that would accelerate the aging process, while remaining below the glass transition temperature of the materials. This approach helps to simulate realistic conditions while avoiding extreme temperatures that could introduce artificial effects. In addition, the aim was to maintain above ambient temperatures to effectively accelerate aging. Consequently, temperatures of 35 °C and 42 °C were chosen to strike a balance between accelerating aging and maintaining relevance for practical applications in offshore wind turbine environments. In addition, the selected temperatures had to meet certain constraints to ensure the reliability of the results. In particular, they had to be within the range of feasible temperatures, with the proviso that they could not exceed the glass transition temperature of the material by more than 10 °C. The glass transition temperature of Sikadur is 50 °C and that of Loctite is 82 °C. This precaution is essential to avoid artificial effects that may not manifest themselves in real or exposed conditions. The purpose is to study the reaction and stability of the adhesive materials in environments to which offshore wind turbines may be exposed. At 22 °C, the adhesives were evaluated in conditions close to their normal use. At 35 °C, a slightly higher temperature, the samples were exposed to high thermal conditions. Finally, at 42 °C, the adhesive samples were tested in more extreme thermal conditions.

However, it is important to note that these tests are not designed to directly predict long-term performance. Instead, they establish a time–temperature equivalency to evaluate the long-term behavior of the adhesive under accelerated conditions. Accelerated testing allows adhesives to be observed more quickly at elevated temperatures, providing insight into their potential behavior over longer periods of time.

Finally, the effect of thermal aging on adhesive properties was analyzed to assess their long-term behavior. This will provide a better understanding of how adhesives maintain their structural integrity under severe environmental conditions. The results of these tests will play a crucial role in optimizing the selection of adhesives for these specific applications.

#### 2.3.2. Multi-Environmental Aging

Multi-environmental aging is an important step in assessing the durability of adhesives. It involves exposing adhesive samples to different environments, including humidity, distilled water, and seawater, in order to simulate a variety of real-world conditions in offshore wind turbines. Distilled water typically has a very low salt content as it is purified by distillation to remove impurities and salts. On the other hand, the seawater used in the aging tests was taken from the Mediterranean Sea and has a conductivity of approximately 37.8 mS·cm^−1^ at 22 °C. In addition, multi-environmental aging will investigate how these different conditions affect the properties of the adhesives, such as mechanical strength, cohesion, resistance to degradation, and other key characteristics.

### 2.4. Gravimetric Measurements

The change in mass of the Sikadur and Loctite samples was measured periodically during aging at room temperature (22 °C) in distilled water, using a 10^−5^ g precision balance, with measurements taken from 1 day up to 1 week intervals.

The formula for the water absorption rate is expressed as a percentage and is given by Equation (1) [23]:

(1) $$M\left(t\right)=\frac{m\left(t\right)-m_{0}}{m_{0}}\times 100,$$

where $M\left(t\right)$ represents the water absorption rate at time t; $m\left(t\right)$ represents the mass at time t; and $m_{0}$ represents the initial mass.

### 2.5. Tensile Tests

Tensile tests were used to evaluate the adhesive’s response to an axial tensile force and to determine its mechanical properties such as tensile strength, tensile modulus, and elongation at break.

Tensile tests are performed on unaged adhesive materials to evaluate their as-received properties. They are also performed on aged adhesive samples. By subjecting the aged adhesive samples to tensile testing, the mechanical behavior of the material can be assessed for its response to exposure to environmental conditions.

Tensile tests were conducted on dumbbell-shaped adhesive specimens following the ASTM D638 standard [2], using a hydraulic testing machine equipped with a 100 kN load cell, at a crosshead displacement speed of 0.5 mm/min and at room temperature.

**Figure 5:**
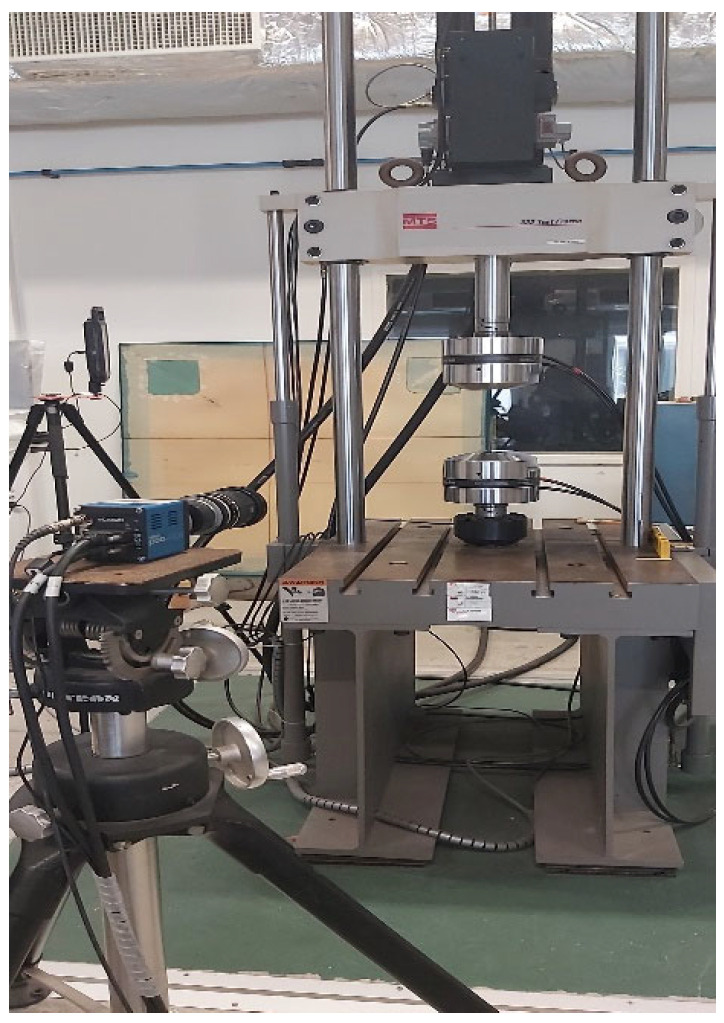
Hydraulic fatigue testing machines (tension/compression) 100 kN + GOM ARAMIS 5 M measurement System 2448 × 2048 pixels 15 Hz with workstation.

In addition, a GOM ARAMIS measurement system, with a remarkable resolution of 2448 × 2048 pixels at 15 Hz, was used to capture and analyze the deformation behavior of the specimens in real time (Figure 5). Finally, it should be noted that three tests were repeated for each configuration in order to obtain the average.

## 3. Results

This section presents the results of all the characterization series carried out on Sikadur and Loctite adhesives. All the data collected during the tensile and aging tests are analyzed in detail, providing valuable information on the performance of these adhesives. In addition, the focus will be on making comparisons between the two adhesives in order to determine their relative suitability for specific marine applications. The implications of these results for the industry and potential areas of research will also be discussed.

### 3.1. Adhesives Behavior before Aging

This section examines the initial mechanical behavior of the Sikadur and Loctite adhesives before they are subjected to the aging process. The results of tensile tests carried out on adhesive samples prior to aging are presented and analyzed in detail.

**Figure 6:**
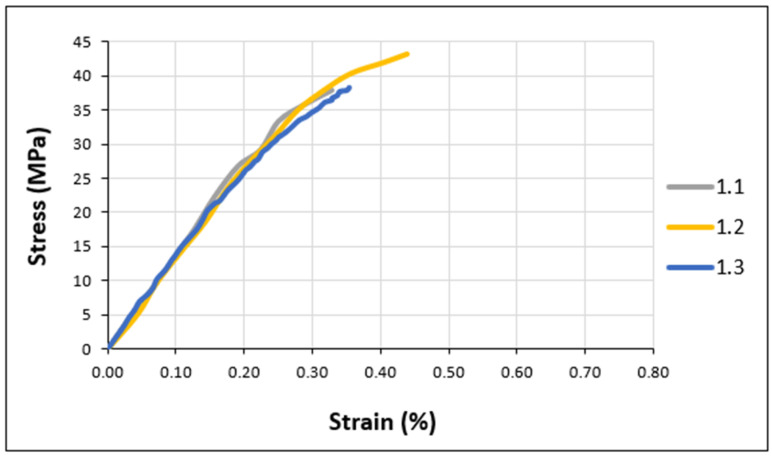
Typical tensile curve (stress–strain) for Sikadur adhesive.

**Figure 7:**
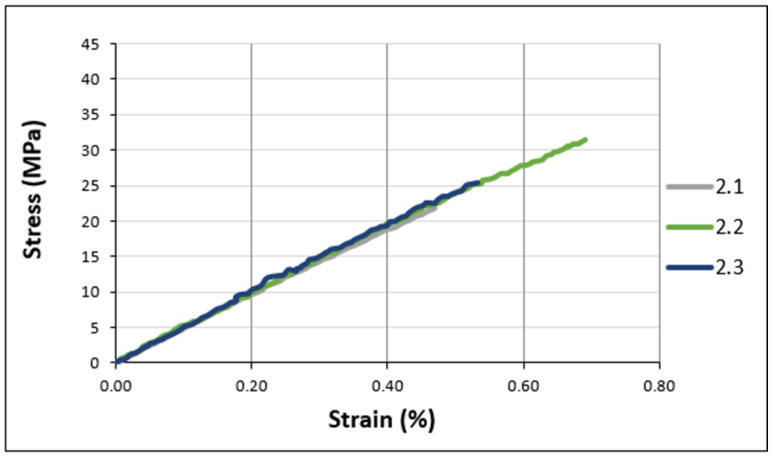
Typical tensile curve (stress–strain) for Loctite adhesive.

Figure 6 and Figure 7 illustrate the tensile behavior of the adhesive samples under various conditions, focusing on the stresses recorded at the point of failure. Superimposed stress–strain curves for unaged Sikadur specimens are shown in Figure 6.

As can be seen from Figure 6 and Figure 7, all the tensile curves show similar trends. This suggests a consistent mechanical behavior under different conditions. In fact, the results show that all the adhesives exhibit elastic behavior with a slight non-linearity observed up to brittle fracture. The assumed elastic character and the non-linearity of the elastic response could be interpreted as a discrete viscoplastic component.

**Figure 8:**
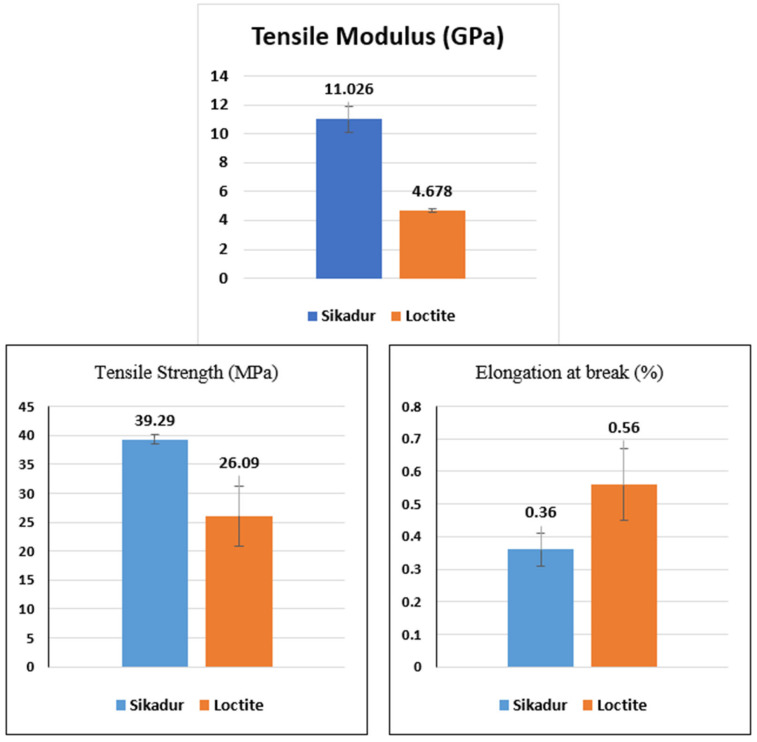
Results of tensile tests on Sikadur 30 and Loctite UK 1351 B25 adhesives.

The experimental tensile properties, extracted from Figure 6 and Figure 7, are summarized in the histograms (Figure 8), including standard deviations for a better understanding.

Specifically, for the Sikadur adhesive, the average failure stress is 39.29 ± 0.84 MPa, and the elastic strain limit is 0.36 ± 0.05%. On the other hand, for the Loctite adhesive, the average ultimate stress reached 26.06 ± 5.17 MPa, with an elastic deformation limit of 0.56 ± 0.11%. These results are very close to the values quoted by the manufacturer.

Previous studies, such as those of Andersen et al. [1] and Gao et al. [9], have highlighted the importance of understanding the performance of adhesives in industrial applications. In addition, Chen et al. [5] investigated the effect of moisture absorption on the mechanical performance of structural adhesive bonds, providing insights that may contribute to the interpretation of the behaviors observed in this study.

These initial mechanical characteristics are used as a reference to evaluate the effect of the aging process on the adhesive materials, following the methodology used in the studies by O’Connor et al. [18] and Williams et al. [19]. This comparative approach provides a better understanding of the performance of Sikadur and Loctite adhesives, and provides information to optimize material selection and maintenance practices in an industrial context.

### 3.2. Water Sorption Kinetics

**Figure 9:**
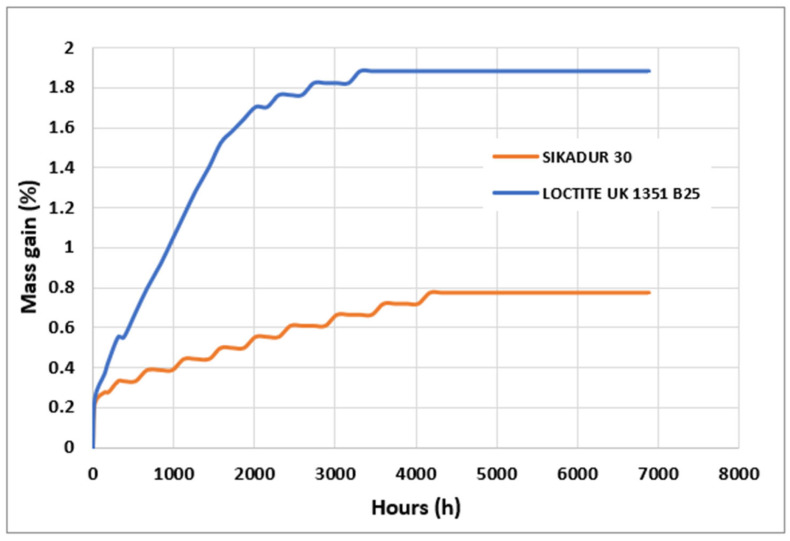
Comparison of experimental mass evolutions of Sikadur 30 and Loctite UK 1351 B25 adhesives during aging at 22 °C in distilled water.

This section focuses on the behavior of the two adhesives under aging conditions. Figure 9 shows the mass uptake (in %) of both adhesives as a function of aging time in the specified environment and temperature. The simulations derived from the absorption model introduced by Bruneaux [4] are superimposed on the experimental results. It is of great importance to emphasize that this model characterizes the absorption of water from the material as a process that is governed by the superposition of two mechanisms: (i) firstly, the Fickian diffusion of water molecules through the free volume of the lattice; (ii) secondly, the reorganization (or relaxation) of the macromolecular chains under the stresses of swelling. This allows the material to absorb more water than Fick’s law alone predicts. This mechanism has been termed viscoelastic diffusion by Berens et al. [24].

The general shape of the mass evolution curves indicates the existence of two apparent regimes in the sorption kinetics.

In the short term and in the early stages, the kinetics of water absorption are very fast. In particular, the Loctite adhesive shows a significant absorption rate in the initial phase and it appears to have a higher free volume occupied by water. This initial phase therefore corresponds mainly to the filling of the free volume by the diffusion of water molecules, which is a thermally activated mechanism [7,25].

In the long term (over longer periods), the increase in water uptake is slow but continuous. No equilibrium is reached, but the evolution becomes asymptotic. This behavior cannot be explained by classical diffusion phenomena and would indicate either the presence of irreversible phenomena within the material or an internal reorganization of the macromolecular structure (chain relaxation) under the effect of swelling stresses [4,24]. Whatever the exact origin of the phenomenon, the observed effect appears to be the long-term non-saturation of the adhesive material.

According to Figure 9, the comparison in the sorption kinetics for the two adhesives immersed in distilled water at 22 °C shows that the initial water uptake is faster for Loctite adhesive. This could be related to a larger free volume accessible to water molecules by diffusion. In the long-term, however, the asymptotic plateau appears to be less pronounced for Loctite than for Sikadur. This result seems to be related to the extraction of the plasticizer during the aging time.

From a quantitative point of view, when examining the slope of the curve between 1000 and 30,000 h, a slope value of 6.5 × 10^−4^ (mass per hour) is observed for the Loctite adhesive and a slope value of 2.5 × 10^−4^ (mass per hour) for the Sikadur adhesive. This means that the Loctite adhesive has much faster absorption kinetics and it absorbs water at a slightly faster rate than the Sikadur adhesive.

Figure 9 also shows an asymptotic plateau, which indicates the point at which water absorption slows down and the material appears to have absorbed as much water as it can under the specific conditions. For Sikadur, this plateau appears to be reached at around 2016 h, whereas for Loctite it appears to be reached at around 2736 h.

Similarly, Figure 9 clearly shows the maximum absorption rates for the two adhesive samples. In fact, the Sikadur adhesive reaches a plateau or equilibrium value at around 0.7%, while for the Loctite adhesive this plateau is reached at around 1.8% by mass.

Furthermore, expressing the changes in mass as a percentage of the initial mass of the samples allows for a comparison of the relative performance of the materials in terms of water absorption. For example, after 2880 h, Sikadur had absorbed approximately 60.77% of its initial mass, while Loctite had absorbed approximately 182.59% of its initial mass.

Finally, taking into account all the results obtained in this section, it can be concluded that there are important implications for the long-term durability and performance of adhesive materials in environments where water absorption is a critical factor.

### 3.3. Adhesive Behavior after Thermal and Hygrothermal Aging (after 46 Days)

As mentioned above, in the dynamic and demanding environment of offshore wind turbines, adhesives play a vital role in ensuring the structural integrity and longevity of critical joints. However, as these installations are subject to a wide range of environmental stressors, including temperature fluctuations and exposure to moisture, the durability of adhesives is of paramount importance. Understanding how adhesives react to prolonged exposure to these conditions is essential to ensure their reliability and performance over time.

This section examines the behavior of adhesives following periods of thermal and hygrothermal aging. This study offers insights into how the two adhesives adapt to the challenges posed by aging. Accelerated aging is employed to identify any alterations in the mechanical properties and structural integrity of the adhesives, with the aim of predicting their long-term behavior.

**Figure 10:**
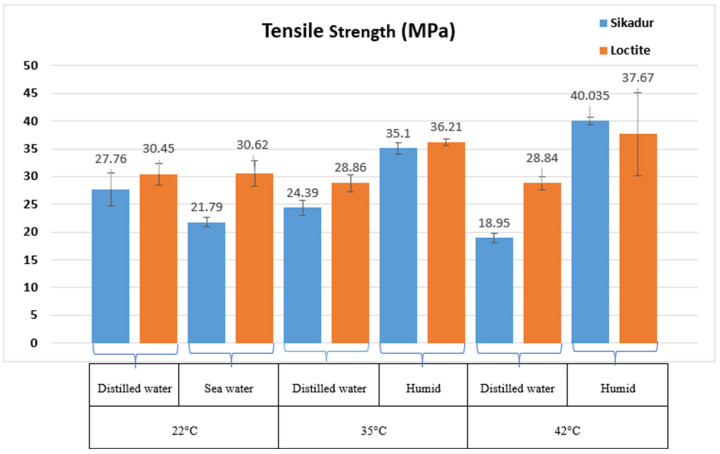
Results of tensile strength of Sikadur 30 and Loctite UK 1351 B25 adhesives after aging for 46 days.

**Figure 11:**
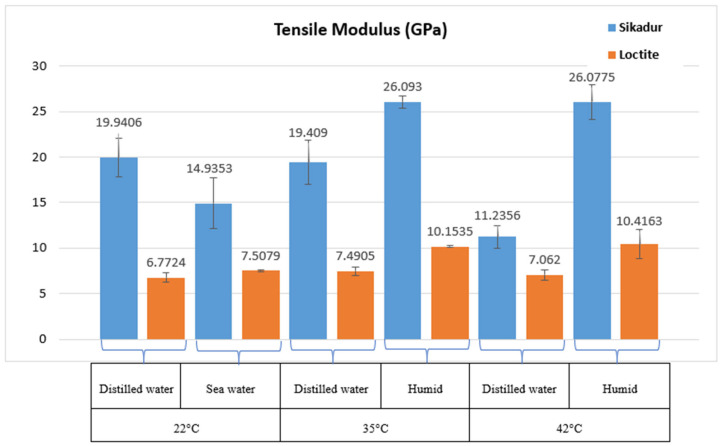
Results of tensile modulus of Sikadur 30 and Loctite UK 1351 B25 adhesives after aging for 46 days.

**Figure 12:**
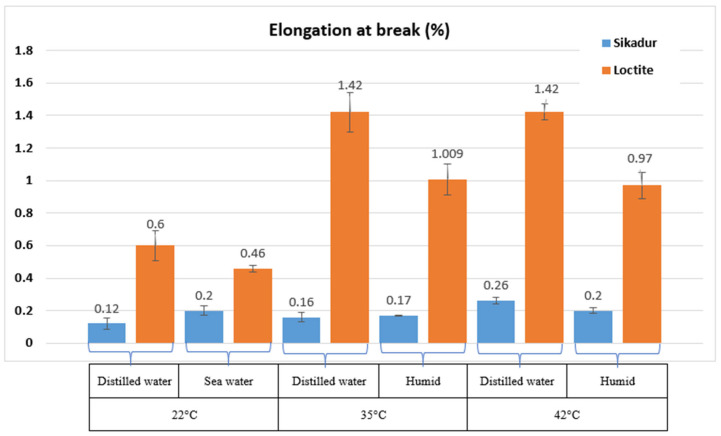
Results of elongation at break of Sikadur 30 and Loctite UK 1351 B25 adhesives after aging for 46 days.

The histograms in Figure 10, Figure 11 and Figure 12 show the tensile behavior of the two adhesives aged at different temperatures and in different environments.

This section focuses on the effect of aging temperature and environment on the tensile behavior of the adhesives studied, as published in many papers on marine adhesives [6,10,13,26]. For this purpose, the adhesive samples were exposed to different conditions—humidity, distilled water, and seawater—coupled with different temperatures (22 °C, 35 °C, and 42 °C) for 46 days. The main results obtained are shown in Figure 10, Figure 11 and Figure 12. The initial parameters, consistent with the initial pre-aging tests, were used, and each temperature condition was evaluated with three samples, in accordance with established standards [8,15,27].

With respect to the temperature range maintained, various changes in the behavior of the adhesives studied were observed, as previously reported in the literature [3,14]. From Figure 10, it can be seen that in a humid environment, Sikadur showed a 9% decrease in yield stress at 35 °C, while at 42 °C it showed a 3% increase. The same tendency was observed for Loctite adhesive, with a 22% increase in stress at 35 °C and a 25% increase at 42 °C. These variations highlight the role of recrystallisation in modifying the stiffness of the polymer matrix [10,16].

A comparison of the parameters in distilled water at different temperatures revealed some interesting information. For the Sikadur adhesive, the stress decreased by 12% between 22 and 35 °C and by 31% between 22 and 42 °C. On the other hand, Loctite adhesive showed an almost stable decrease of 5% between 22 and 35 °C and remained constant between 22 and 35 °C and between 22 and 42 °C. These comparative observations are consistent with those reported in published work on the effect of temperature on the mechanical properties of adhesives [7,17].

In the case of Sikadur adhesive, the main observations correlate well with thermomechanical principles [16,28]. An increase in temperature resulted in an increase in deformation (Figure 12), indicating the increased ductility of the material under elevated thermal conditions. Sikadur adhesive exhibited its highest tensile strength (40.035 MPa) at 35 °C in a humid environment, highlighting its suitability for demanding conditions [1].

In terms of material stiffness, Figure 11 shows that the tensile modulus of the material reaches its maximum at 35 °C (26,093 MPa), indicating that the material effectively maintains its stiffness. The superior performance in distilled water compared to seawater is consistent with Refs. [11,29]. It also appears that the presence of moisture improves the performance, particularly at higher temperatures, suggesting a beneficial interaction with moisture. A similar trend was also observed for Loctite adhesive. In fact, as the temperature increases, the material deformation (ductility) becomes more important, which is the case for Sikadur. Loctite adhesive reached its maximum tensile strength (37.67 MPa) at 42 °C in a humid environment, indicating remarkable strength, particularly at elevated temperatures. The tensile modulus reached its maximum at 35 °C (10,153.5 MPa), demonstrating excellent stiffness.

It should be noted that when seawater is combined with the positive influence of humidity on performance, the behavior of Sikadur is well illustrated.

In terms of comparative evaluation, both Loctite and Sikadur adhesives showed excellent mechanical properties under various conditions, confirming the findings of recent work in similar areas of research [6,30,31].

### 3.4. Adhesives Behavior at Longer Periods

**Figure 13:**
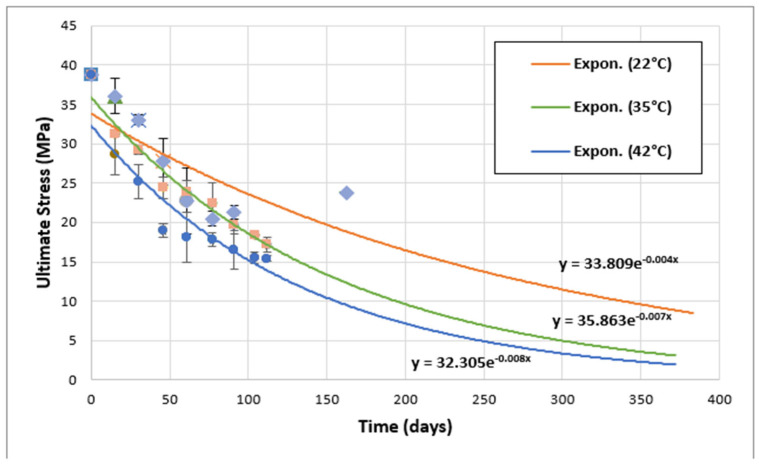
Evolution of ultimate tensile strength (UTS) over time for Sikadur adhesive.

**Figure 14:**
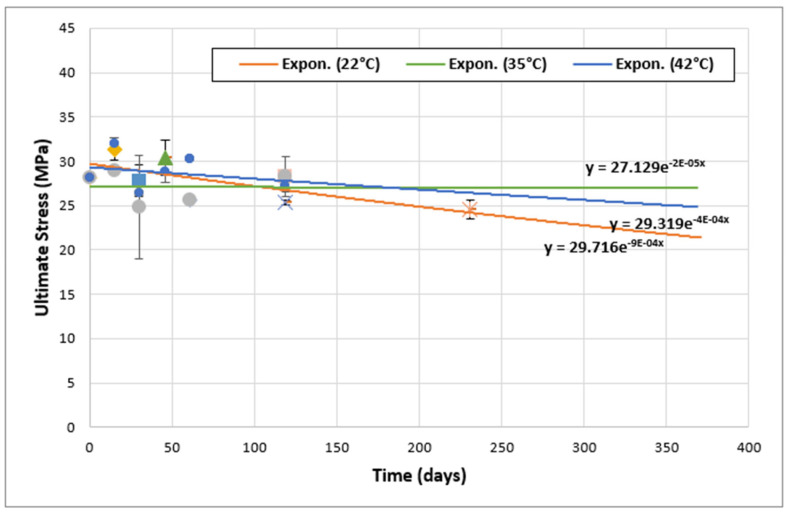
Evolution of ultimate tensile strength (UTS) over time for Loctite adhesive.

According to previous published work [9,32], the comparison between the two adhesives should consider all aspects in order to make the right decisions. For this purpose, in this section, the variation in the ultimate tensile strength (UTS) for both adhesives at different aging times and at different temperatures (22, 35, and 42 °C) has been followed. Then, in a second phase, this section deals with the detailed description of the evolution of the mechanical behavior of the two adhesives, especially at longer aging times. The results obtained are shown in Figure 13 and Figure 14 for Sikadur and Loctite adhesives, respectively.

As can be seen in Figure 13 and Figure 14, this study of the mechanical properties of aged Sikadur (up to 163 days) and Loctite (up to 231 days) shows a clear trend towards a progressive decrease in ultimate tensile strength (UTS) with the exposure time. Such a trend can take the form of an exponential function, as follows:

(2) $$\sigma =ae^{-kt},$$

where σ represents the ultimate stress; t is the aging time; and a and k are parameters. The correlation between our observations and those reported in other publications on the aging of adhesives utilized in marine environments is noteworthy [12,19,33].

For Sikadur adhesive exposed in distilled water at 22 °C, the UTS decreased from an initial value of 38.75 MPa to 23.67 MPa after 163 days. A similar trend was observed for exposure at 35 °C and 42 °C. This progressive degradation is consistent with the expected effects of aging. This also highlights the importance of regular maintenance for adhesive materials during their service life [18,20].

Loctite adhesive also shows a similar aging behavior. In fact, at 22 °C, the UTS of Loctite adhesive decreased from 28.12 MPa to 24.56 MPa after 231 days. Similar trends were observed at 35 °C and 42 °C, indicating the evolution of the mechanical properties of the adhesive material over time [34,35,36].

Based on Figure 13 and Figure 14, all parameters describing the aging behavior of the two adhesives were extracted and then summarized in Table 3.

Comparing Sikadur and Loctite, it appears that Loctite generally retains a higher tensile strength under similar conditions, indicating potential resistance to the effects of aging. However, both adhesives show significant changes in UTS over time. In fact, both Sikadur and Loctite adhesives undergo some degree of degradation over time. Furthermore, this degradation is more pronounced at elevated temperatures which accelerate the aging process [2,27].

The exponential trends also highlight the need for a better understanding of the aging behavior of adhesives [20,36]. This fact underlines the essential role of regular evaluation and maintenance in order to ensure the long-term reliability of adhesives [17,20,32].

**Figure 15:**
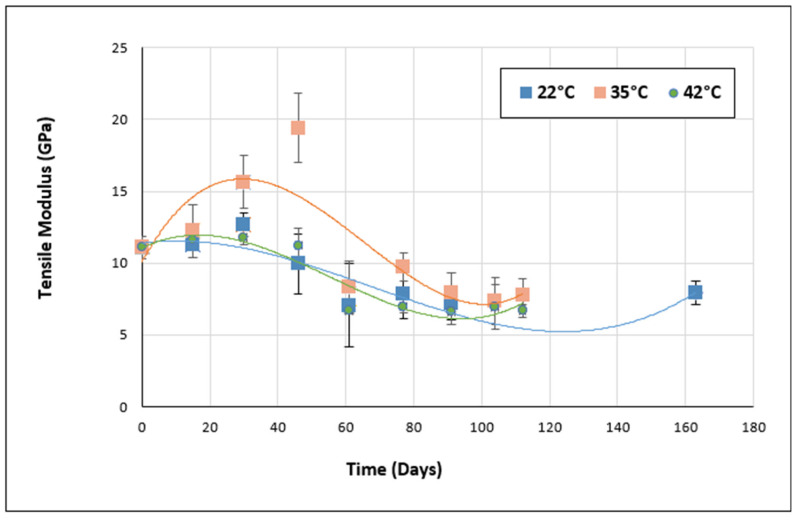
Evolution of tensile modulus over time for Sikadur adhesive.

**Figure 16:**
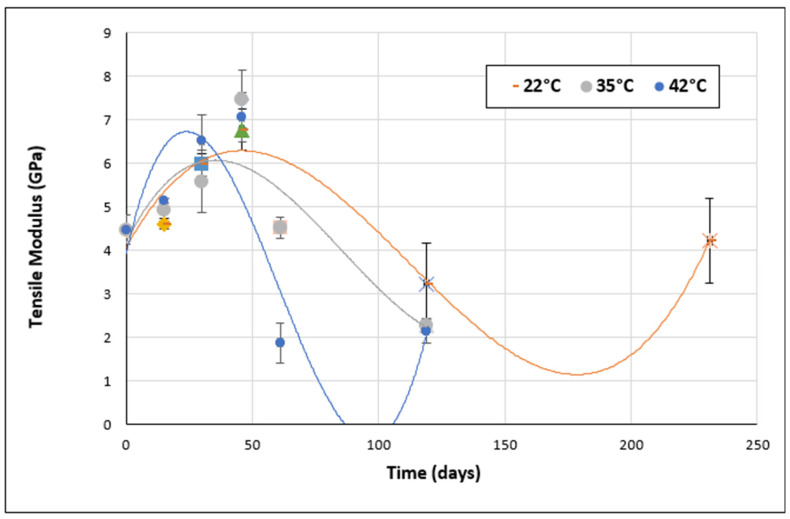
Evolution of tensile modulus over time for Loctite adhesive.

On the other hand, in addition to the tensile strength, the stiffness of the aged materials was also evaluated during the aging period. The evolution of the tensile modulus over time for Sikadur and Loctite adhesives is shown in Figure 15 and Figure 16, respectively.

From Figure 15 and Figure 16, it can be seen that for both Sikadur and Loctite adhesives, the variation in tensile modulus over time shows several trends.

Firstly, for the Sikadur adhesive exposed at 22 °C, an initial increase in tensile modulus was observed during the first 30 days of aging, followed by a gradual decrease up to 77 days. Thereafter, the tensile modulus appears to stabilize. This trend may indicate an initial phase of aging during which chemical bonds are strengthened, followed by a possible gradual deterioration of mechanical properties.

At 35 °C, there is a significant increase in the tensile modulus during the first 30 days of aging, suggesting a more rapid response at higher temperatures. However, after this period, there is a significant decrease in the modulus, which could indicate an early deterioration of mechanical properties at elevated temperatures.

For samples exposed at 42 °C, a progressive decrease in tensile modulus was observed throughout the aging process. This again confirms the continuous degradation of mechanical properties with increasing temperature.

In the case of Loctite adhesive, an initial increase in tensile modulus was observed during the first 30 days (at 22 °C), followed by a gradual decrease. This suggests a period of initial cure followed by subsequent degradation. At 35 °C, an initial increase is observed, but this is followed by a marked decrease, indicating an early degradation of mechanical properties at higher temperatures. Finally, at 42 °C, a similar trend of gradual decrease in tensile modulus is observed, suggesting continuous degradation at elevated temperatures.

In general, when comparing the performance of Sikadur and Loctite adhesives, Loctite appears to be more resistant to mechanical degradation at higher temperatures. In fact, Loctite adhesive maintains relatively higher tensile moduli than Sikadur adhesive under the same conditions.

However, it is worth noting that adhesive performance can be influenced by various factors, such as chemical composition, molecular structure, and environmental conditions. Further analysis would therefore be required to fully understand the results obtained.

**Figure 17:**
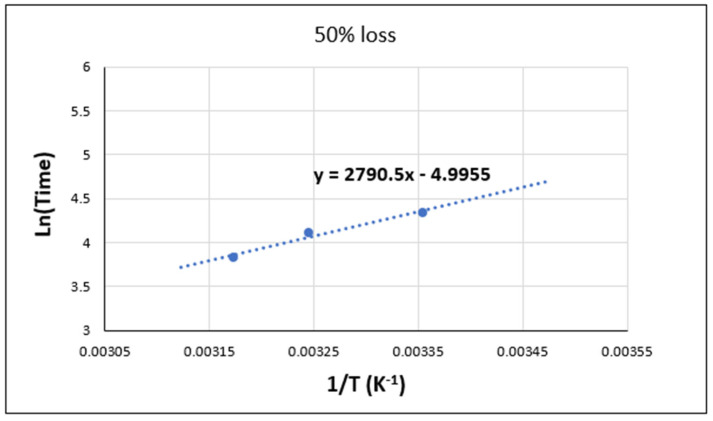
Validation of the Arrhenius law for Sikadur adhesive in distilled water.

**Figure 18:**
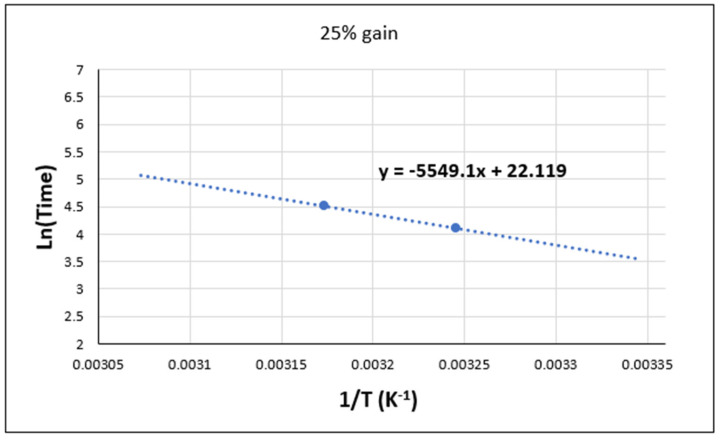
Validation of the Arrhenius Law for Sikadur adhesive in humid environment.

The following section focuses on the limitations of the Arrhenius law in the case of the two adhesives when exposed to an aging environment. To achieve this, the logarithm of the aging times (*t*) is plotted against the reciprocal of the temperature (1/*T*) in Figure 17 and Figure 18.

As can be seen in Figure 17, the aging of Sikadur adhesive shows a remarkable validation of the Arrhenius law, as it shows a linear relationship between the logarithm of time (in days) and the reciprocal of the absolute temperature (in Kelvin), as in Equation (3):

(3) $$Ln\left(t\right)=-\frac{E_{a}}{R}\times \frac{1}{T}+\ln(A)$$

where t is the aging time; $E_{a}$ is the activation energy; R is the perfect gas constant; T is the absolute temperature; and A is the pre-exponential factor.

The results obtained are consistent with recent studies on adhesives used in marine environments [11,14,26]. In particular, the linear correlation between Ln(time) and 1/*T* (Figure 17) indicates that the aging process of Sikadur adhesive follows Arrhenius kinetics [37]. This result provides a mathematical model for predicting the behavior of the adhesive over time under different temperature conditions.

In addition, the curve obtained in Figure 17 also shows a 50% loss of properties, which represents a significant reduction in the mechanical performance of the adhesive [28]. This can give an indication of the lifetime of the material and its ability to resist.

On the other hand, the curve obtained in Figure 18 shows an increase in the mechanical performance of the adhesive as the wet temperature increases. In fact, at higher temperatures, the chemical reactions responsible for polymerization and curing of the adhesive can take place more rapidly. This makes it possible to improve its structure and mechanical properties.

It is, however, essential to acknowledge that this interpretation is contingent upon an empirical relationship. Other factors, such as water, humidity, and environmental composition, may also influence the mechanical performance of adhesive materials.

By applying the Arrhenius law (Equation (3)) and utilizing the provided data, the long-term behavior of the Sikadur adhesive under these specific conditions can be estimated. Indeed, by extrapolating the Ln(days) values for additional values of (1/T), the evolution of the aging time in these environments can be predicted.

However, it should be noted that long-term extrapolation may be subjected to uncertainties and limitations. Indeed, the extrapolation is based on limited experimental data and on the assumption of continuity of the observed behavior in the available data. In addition, other environmental and aging factors could potentially affect adhesive performance over time. As a result, long-term projections may be subject to potential variation.

## 4. Discussion

The results of the extensive experimental program shed light on the performance of Sikadur and Loctite adhesives in offshore wind applications, and provided important insights that can be interpreted in the context of previous studies and working hypotheses.

Comparing the results of this study with existing research in the field of adhesive behavior, it is clear that both Sikadur and Loctite adhesives exhibit important mechanical properties. These results are consistent with previous studies that have highlighted the importance of durable and resilient adhesives to withstand the harsh conditions encountered in offshore environments.

In addition, the differences observed in water absorption kinetics between the Sikadur and Loctite adhesives add to the body of knowledge on adhesive behavior under varying environmental conditions. By understanding these differences, engineers and researchers can make more informed decisions when selecting adhesives for specific offshore wind turbines projects, taking into account factors such as initial absorption rates and maximum absorption capacities.

The implications of the findings extend beyond the specific materials tested in this study, providing important insights into the broader challenges and opportunities in offshore wind applications. By considering the implications of these results in the broadest possible context, researchers can identify areas for further investigation and improvement in adhesive technology for offshore wind structures.

Future research directions may include refining predictive models for assessing the long-term performance of adhesives, incorporating additional environmental factors such as UV exposure and mechanical loading. In addition, studies focusing on the development of novel adhesive formulations optimized for offshore wind turbines applications could further advance the field and contribute to the sustainability and reliability of renewable energy sources.

## 5. Conclusions

In this work, an extensive experimental program was carried out at the LMA laboratory in Marseille in order to evaluate the durability performance of two adhesives and to determine their suitability for offshore wind applications. The investigation included a series of mechanical tests and accelerated aging simulations (in terms of temperature, relative humidity (RH), aging times).

The following main conclusions can be drawn from the studies carried out on Sikadur and Loctite adhesives under different aging conditions:

- Both adhesives showed significant mechanical properties, demonstrating their potential for this industrial use.
- Loctite adhesive has a slightly faster initial absorption rate than Sikadur adhesive, but the latter reaches an asymptotic plateau at a lower maximum absorption rate than Loctite adhesive.
- When subjected to aging conditions, Sikadur adhesive showed good tensile strength, particularly at elevated temperatures and in humid environments. Loctite adhesive also showed good mechanical properties, with notable resistance observed at higher temperatures and in wet conditions.
- When both adhesives were exposed to longer periods of time, the aging results showed a progressive deterioration in the mechanical properties as a function of aging time. This appears to follow an exponential function.
- The aging results show a clear correlation with the Arrhenius law, providing a predictive tool for the aging process. The aging process thus follows Arrhenius kinetics.

In addition to confirming the results obtained during this investigation, it is imperative to emphasize the innovative aspects of the methodology employed in comparison to existing research paradigms. This study integrates a comprehensive experimental program encompassing mechanical tests and accelerated aging simulations to assess the durability performance of two adhesives for offshore wind applications. The incorporation of these diverse approaches yields valuable insights into the mechanical properties and aging behavior of the adhesives, as well as a predictive framework for evaluating their service life. Of particular significance is the correlation with the Arrhenius law, which represents a significant advance in comprehending the long-term performance of adhesives in offshore wind turbine applications. Further research could be conducted with the objective of refining predictive models to encompass additional environmental factors and operational conditions. This would enhance the understanding of material behavior and optimize the design and maintenance of offshore wind structures.

## Figures and Tables

**Table 1:** Main characteristics of Sikadur 30 adhesive (SIKA France SAS).

Property	Symbol	Value (MPa)
Tensile modulus	E_t_	9600
Tensile strength	σ_t_	14–17 (at +15 °C) and 16–19 (at +35 °C)
Compressive modulus	E_c_	11,200
Compressive strength	σ_c_	24–27 (at +15 °C) and 26–31 (at +35 °C)
Shear strength	τ	14–17 (at +15 °C) and 16–19 (at +35 °C)
Tensile modulus	E_t_	9600
Tensile strength	σ_t_	14–17 (at +15 °C) and 16–19 (at +35 °C)

**Table 2:** Characteristics of Loctite UK 1351 B25 (Henkel Adhesive Technologies, Düsseldorf, Germany).

Property	Symbol	Value (MPa)
Tensile strength	σ_t_	26
Compressive modulus	E_c_	4740
Compressive strength	σ_c_	71

**Table 3:** Parameters of exponential decrease in ultimate tensile strength (UTS) as function of time.

	*T* (°C)	*a*	*k*
Sikadur adhesive	22	32.305	0.008
	35	35.863	0.007
	42	33.809	0.004
Loctite adhesive	22	29.716	9.00 × 10^−4^
	35	27.129	0.20 × 10^−4^
	42	29.319	4.00 × 10^−4^

## Data Availability

Data are contained within the article.
